# External apical root resorption in maxillary root-filled incisors after orthodontic treatment: A split-mouth design study

**DOI:** 10.4317/medoral.17586

**Published:** 2011-12-06

**Authors:** José M. Llamas-Carreras, Almudena Amarilla, Eduardo Espinar-Escalona, Lizett Castellanos-Cosano, Jenifer Martín-González, Benito Sánchez-Domínguez, Francisco J. López-Frías

**Affiliations:** 1 Associate Professor, Department of Orthodontics, School of Dentistry, University of Seville, C/ Avicena s/n, 41009-Seville, Spain; 2 Doctoral fellow, Department of Orthodontics, School of Dentistry, University of Seville, C/ Avicena s/n, 41009-Seville, Spain; 3 Professor, Department of Stomatology, School of Dentistry, University of Seville, C/ Avicena s/n, 41009-Seville, Spain

## Abstract

Introduction: The purpose of this study was to compare, in a split mouth design, the external apical root resorption (EARR) associated with orthodontic treatment in root-filled maxillary incisors and their contralateral teeth with vital pulps. Methodology: The study sample consisted of 38 patients (14 males and 24 females), who had one root-filled incisor before completion of multiband/bracket orthodontic therapy for at least 1 year. For each patient, digital panoramic radiographs taken before and after orthodontic treatment were used to determine the root resortion and the proportion of external root resorption (PRR), defined as the ratio between the root resorption in the endodontically treated incisor and that in its contralateral incisor with a vital pulp. The student’s t-test, chi-square test and logistic regression analysis were used to determine statistical significance. Results: There was no statistically significant difference (p > 0.05) between EARR in vital teeth (1.1 ± 1.0 mm) and endodontically treated incisors (1.1 ± 0.8 mm). Twenty-six patients (68.4%) showed greater resorption of the endodontically treated incisor than its homolog vital tooth (p > 0.05). The mean and standard deviation of PPR were 1.0 ± 0.2. Multivariate logistic regression suggested that PRR does not correlate with any of the variables analyzed. Conclusions: There was no significant difference in the amount or severity of external root resorption during orthodontic movement between root-filled incisors and their contralateral teeth with vital pulps.

** Key words:**Endodontics, orthodontics, root canal treatment, root resorption.

## Introduction

External apical root resorption (EARR) is a common event observed in association with orthodontic movement (1,2). It can begin in the early levelling stages of orthodontic treatment (3). Orthodontic EARR is considered as a type of surface resorption (also called repair related resorption), caused by damage to cementum as a result of mechanical dental trauma, surgical procedures, orthodontic forces, or excessive pressure of impacted tooth or tumours (4). Morphologically and radiographically is characterized by apical rounding, however, it can show different degrees, from a slightly blunted or round apex to a grossly resorbed apex (5). Mean values of EARR ranging from 0.5 to 5 mm of root shortening have been reported (6,7).

The specific causes of orthodontic EARR are not well understood (8), but it appears that results from a combination of mechanical and biological factors (9). The following are amongst the mechanical factors: extensive tooth movement, root torque and intrusive forces, movement type, orthodontic force magnitude, duration and type of force (10). The use of strong forces, especially intrusive or tipping forces and prolonged treatment is directly related to an increase in root resorption associated with orthodontics (1,9). Evidence suggests that comprehensive orthodontic treatment causes increased incidence and severity of root resorption, and heavy forces might be particularly harmful (8). The biological factors include the following: individual susceptibility, genetic influence, systemic factors (hormone unbalance and endocrine disturbances), anatomical factors, teeth agenesis and medication intake (9-11). Levander & Malmgren (12) indicated that teeth with blunt or pipette shaped roots of maxillary central incisors were at greater risk for EARR than teeth with normal root form.

Is the extent of orthodontic EARR the same on teeth with vital pulps as endodontically treated teeth? The common sense of many professionals is that endodontically treated teeth are more susceptible to EARR, although they respond similarly to vital teeth to orthodontic forces (5).

EARR during orthodontic treatment is considered an unwanted consequence of orthodontic treatment that could result in loss of tooth structure. The maxillary incisors most commonly show EARR after orthodontic treatment and are used to determine root resorption during experimental studies (13). Remington et al. (14) reported that the maxillary incisors underwent root resorption more frequently and to a greater degree than the rest of the teeth during orthodontic treatment. It has been shown that when there is no root resorption of the maxillary or mandibular incisors, resorption of other teeth is improbable (2,15).

The purpose of this study was to compare, in a split mouth design, EARR associated with orthodontic treatment in root-filled maxillary incisors and their contralateral teeth with vital pulps. The null hypothesis was that there is no difference between root resorption in endodontically treated teeth and their control teeth with vital pulps.

## Material and Methods

In a split-mouth design, EARR was determined in root-filled maxillary incisors and their contralateral homolog teeth with vital pulps. The ethic committee of the School of Dentistry approved the study and all the patients included gave written informed consent.

Sample

Sample size (n = 38) was based on a power calculation (power 0.90; p = 0.05). Inclusion criteria were as follows: 1) to have a root-filled maxillary incisor before placement of orthodontic bands; 2) to have the contralateral homologous incisor without endodontic treatment and with vital pulp, assessed with thermal test; 3) to complete multiband/bracket orthodontic therapy with duration of active treatment exceeding 1 year; and 4) to show incisal integrity throughout the active treatment period evaluated in the pretreatment and post-treatment orthodontic study casts. Patients with fractured incisors or otherwise mutilated roots and teeth with radiological signs of periapical pathosis in the pretreatment radiography (Periapical Index score of 3, 4 or 5) (16) were excluded. Fourteen males and 24 females, aged 30.7 ± 10.2 years, with duration of active treatment 24.0 ± 12.0 months, were randomly recruited.

Radiographic examination and evaluation

For each patient, digital panoramic radiographs, taken before and after orthodontic treatment, were used. Two trained radiology assistant using a digital ortho-pantomograph machine (Promax®, Planmeca, class 1, type B, 80 KHz, Planmeca, Helsinki, Finland) took the panoramic radiographs. The same radiology assistant and panoramic machine were used for the two radiographs of each patient. A mirror and horizontal and vertical light guides, incorporated into the machine, facilitated standardization of head positioning. Images were obtained using the Dimaxis Pro 3.1.1 program (Planmeca Group). In all cases radiographs showed the entire corono-apical length of measured teeth with the apex clearly defined. Digital panoramic images were displayed in a 17” Plug and Plag model monitor using a NVIDIA Riva TNT 2 model 64 graphic card with 32 bit quality colour and 1280x1024 pixels resolution (120 ppp) in a room with subdued light.

Measurements were made before and after orthodontic treatment using Adobe Photoshop CS® software. Root resorption in the endodontically treated tooth (RRE) and contralateral tooth with vital pulp (RRV) were calculated in millimetres as described previously (17). Briefly, radiographs were standardized by measuring the greatest distance from incisal edge to cementoenamel junction on each patient's in pretreatment and post-treatment radiographs. Crown lengths and root lengths in the initial and final radiographs were calculated. In order to allow intra-patient standardization, root resorptions in the root filled tooth (RRE) and contralateral tooth with vital pulp (RRV) were calculated. Then, the proportion of root resorption (PRR) for each patient was calculated as follows: PRR = RRE / RRV.

Statistical analysis

Student t-test for matched pairs and logistic regression analysis were applied. The level for statistical significance was set at p < 0.05.

## Results

Of the 38 patients selected, 39.5% (n = 15) had Class I, 39.5% (n = 15) had Class II, and 21.0% (n = 8) Class 3 occlusions. The characteristics of the patients and orthodontic treatments, mean and standard deviation of EARR found both in endodontically treated teeth (RRE) and in their contralateral vital teeth (RRV), expressed in millimetres, and the proportion of root resorption (PPR) of the total sample are listed in ( Table 1). Although the vital teeth showed, on average, a slightly degree of mean EARR, there was no statistically significant difference (p > 0.05) between EARR in untreated teeth (RRV = 1.1 ± 1.0 millimetres) and endodontically treated incisors (RRE = 1.1 ± 0.8 millimetres). However, comparing the two analyzed teeth in the same subject, twenty-six patients (68.4%) showed greater resorption of the root filled incisor than its homolog vital tooth (PRR > 1). In twelve patients (31.6%), the control teeth with vital pulps resorbed largely (PRR < 1). The mean and standard deviation of PPR were 1.0 ± 0.2, indicating that, in the total sample, there was no significant differences between the amounts of root resorption in the endodontically treated incisors and its contralateral control teeth with vital pulps.

**Table 1:**
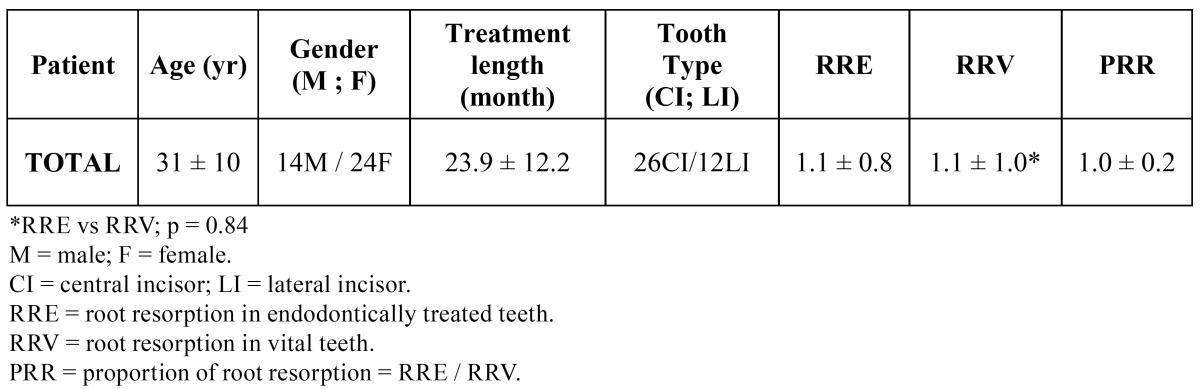
Characteristics of the patients, orthodontic treatments, root resorption (mm) and proportion of root resorption. Results are expressed as mean ± standard deviation.

Multivariate logistic regression was run with gender (0 = female; 1 = male), age (yr), treatment length (months), type of treatment (0 = without extractions; 1 = with extractions), and tooth type (0 = central; 1 = lateral) as independent explanatory variables, and “proportion of root resorption” (dichotomized: 0 = PRR < 1; 1 = PRR > 1) as the dependent variable ( Table 2). The analysis suggested that PRR does not correlate with any of the variables.

**Table 2:**
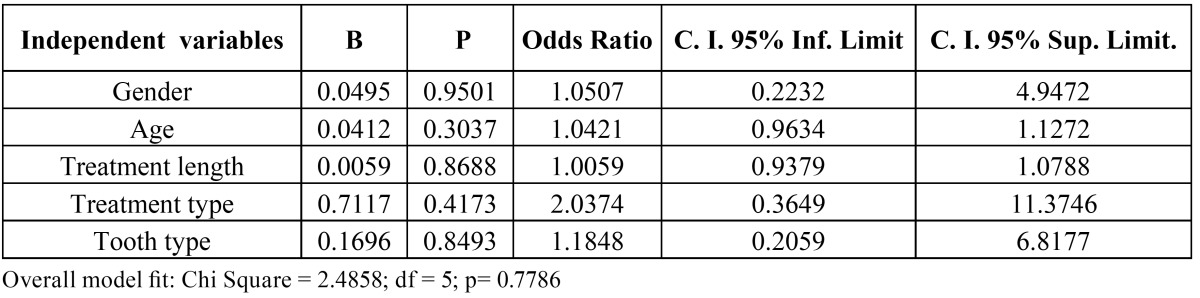
Multivariate logistic regression analysis of the influence of the independent variables gender (0 = female; 1 = male), age (yr), treatment length (months), type of treatment (0 = without extractions; 1 = with extractions), and tooth type (0 = central; 1 = lateral) on the dependent variable “proportion of root resorption” (0 = PRR < 1; 1 = PRR > 1).

## Discussion

Many general dentists and other dental specialists believe that EARR is avoidable and hold the orthodontist responsible when it occurs during orthodontic treatment (18). Histological studies reported greater than a 90% occurrence of EARR in orthodontically treated teeth (19). With panoramic or periapical radiographs, EARR is usually less than 2.5 mm (8,18). In the present study, pre- and post-treatment tooth lengths of the maxillary incisors were measured on panoramic radiographs of 38 subjects. Previous studies have also used panoramic radiographs in assessing external root resorption during orthodontic movement (20,21). In general, extraoral radiographs are considered less accurate than periapical radiographs in studying the extent of EARR. The use of panoramic films to measure pre- and post-treatment root resorption could overestimate (22) or underestimated (23) the amount of root loss after orthodontic tooth movement.

It has been demonstrated that an important source of error associated with panoramic radiographs is head positioning with respect to tilting. Stramotas et al. (24) concluded that linear measurements on panoramic radiographs taken at different times are sufficiently accurate if the occlusal plane is positioned similarly on the 2 occasions and the extent of tilting does not exceed 10°. In this study, the same radiology assistant and panoramic machine were used for all radiographs, and a mirror, and horizontal and vertical light guides, incorporated into the machine, facilitated standardization of head positioning. Moreover, the objective was to compare the EARR in both teeth groups rather than to determine the absolute values of root lost. The measurement of root resorption in this study has been performed taking into account the length of the crown. Thus, root resorption was calculated as “standardized root resorption”, being a more accurate measure than those used in other studies (21).

The present study revealed that, in the total sample, there were not significant differences between the amounts of EARR in the root filled teeth and their contralateral teeth with vital pulps. These results are in accordance with several studies carried out using animal models. Huettner et al. (25) observed similar root resorption in both groups evaluating the root structure of monkey teeth with both vital and non-vital pulps (root canal treatment) following orthodontic movement. Also in animal models, Mah et al. (26) found no significant differences between external root resorption of root filled teeth and teeth with vital pulps when both were subjected to orthodontic forces.

The results of the present study are also in agreement with several studies performed in humans. Esteves et al. (13), analyzing 16 patients who had a maxillary central incisor treated endodontically before initiation of the orthodontic movement, and a vital homologous tooth (for control), did not find statistically significant differences in EARR (p>0.05). However, the power of these studies was low because the samples consisted of only eighteen and sixteen patients, respectively, who had completed orthodontic therapy. A sample of this size is usually considered too small for statistical significance. Llamas-Carreras et al. (17) analyzing 71 patients, did not find significant differences in the amount or severity of orthodontic EARR between root filled teeth and their homolog teeth with vital pulps.

On the contrary, Spurrier et al. (27) studied forty-three patients who had one or more root filled incisors before orthodontic treatment and who exhibited signs of EARR after treatment. Results showed that incisors with vital pulps resorbed to a significantly greater degree than incisors that had been root filled. Mirabella & Årtun (6), in a sample of 39 pairs of contralateral teeth with and without root canal treatment in 36 patients, found that there was significantly less resorption in root filled teeth.

Maxillary incisors with a previous history of trauma have a higher susceptibility to pulpal complications during orthodontic treatment are more susceptible to orthodontic EARR than healthy control teeth (4). However, there is a lack of randomized clinical trial data about patients with previously traumatized teeth with EARR before orthodontic treatment. Observational data indicate a greater chance that orthodontic movement will enhance the resorptive process in this situation (5,28). Wickwire et al. (29) in a retrospective study reviewed 45 orthodontic patient case histories that contained 53 endodontically treated teeth. They found that appeared to be greater radiographic evidence of root resorption in the endodontically treated teeth compared to those with vital pulps. In the present study, some of the maxillary incisors included in the study had previous history of trauma, although the extent to which either tooth, root-filled incisors as well as incisors with vital pulps, may have been traumatized was not known. This could explain, at least in part, that, when comparing the two analyzed teeth in the same subject, twenty-six patients (68.4%) showed greater resorption of the endodontically treated incisor than its homolog with vital pulp, and only in twelve patients (31.6%) the control vital tooth resorbed largely. A previous study (17) found a greater PRR (resorption in endodontically treated tooth / resorption in contralateral vital tooth) during orthodontic movement in incisors compared to others tooth types. The cause of the root canal treatment could explain in part these results: all incisors were treated endodontically because of dental injury, but the other teeth (canines, pre-molars and molars) were endodontically treated teeth because of tooth decay.

The multivariate logistic regression analysis indicated that EARR was not associated to age, gender, treatment length, type of treatment (with or without extractions) nor tooth type (p > 0.05). In accordance, Jiang et al. (30) found no difference in the incidence or severity of resorption between male and female patients. However, Spurrier et al. (27) reported that, when the sample was subdivided by gender, no significant difference in the amount of root resorption of the endodontically treated teeth was evident, but the male patients exhibited a greater degree of change in the control teeth.

## Conclusions

There was no significant difference in the amount or severity of external apical root resorption during orthodontic movement between traumatized and endodontically treated incisors and their contralateral vital control teeth.
